# Diagnostic and prognostic role of circular RNAs: Analytical challenges and emerging opportunities

**DOI:** 10.1016/j.isci.2026.116929

**Published:** 2026-08-21

**Authors:** Alessandra Glovi, Panagiota M. Kalligosfyri, Antonella Miglione, Gabriella Iula, Sima Singh, Michelino De Laurentiis, Stefano Cinti

**Affiliations:** 1 Clinical and Translational Oncology, Scuola Superiore Meridionale, 80138 Naples, Italy; 2 Department of Pharmacy, University of Naples Federico II, 80131 Naples, Italy; 3 Department of Public Health, University Federico II of Naples, 80131 Naples, Italy; 4 Genomic and Experimental Medicine, Scuola Superiore Meridionale, 80138 Naples, Italy; 5 Department of Breast and Thoracic Oncology, Istituto Nazionale Tumori IRCCS “Fondazione G. Pascale”, Naples, Italy; 6 Sbarro Institute for Cancer Research and Molecular Medicine, Center for Biotechnology, College of Science and Technology, Temple University, Philadelphia, PA 19122, USA; 7 Department of Chemistry, Faculty of Science, Chulalongkorn University, Bangkok 10330, Thailand

**Keywords:** circRNA, back-splice junction, nucleic acid sensing, diagnosis, prognosis

## Abstract

Circular RNAs (circRNAs) are covalently closed non-coding RNAs characterized by high stability, tissue-specific expression, and important regulatory roles in gene expression and cellular pathways across cancer, neurodegenerative, cardiovascular, autoimmune, and infectious diseases. Their stability in biofluids and disease-associated expression patterns make them attractive candidates for diagnostic and prognostic biomarkers. This review examines the analytical strategies used to detect and quantify circRNAs, including amplification-based methods (RT-qPCR, droplet digital PCR, and isothermal amplification), hybridization-based assays (Northern blot, FISH, and microarrays), sequencing approaches (short- and long-read RNA-seq), and emerging biosensor technologies. These methods are discussed in the context of their principles, strengths, limitations, and suitability for different research and clinical applications. By integrating current knowledge of circRNA biology with practical guidance on analytical workflows, this review provides a framework for selecting appropriate detection strategies and supports the development of reliable circRNA-based diagnostics and future precision medicine applications.

## Graphical abstract

**Figure:**
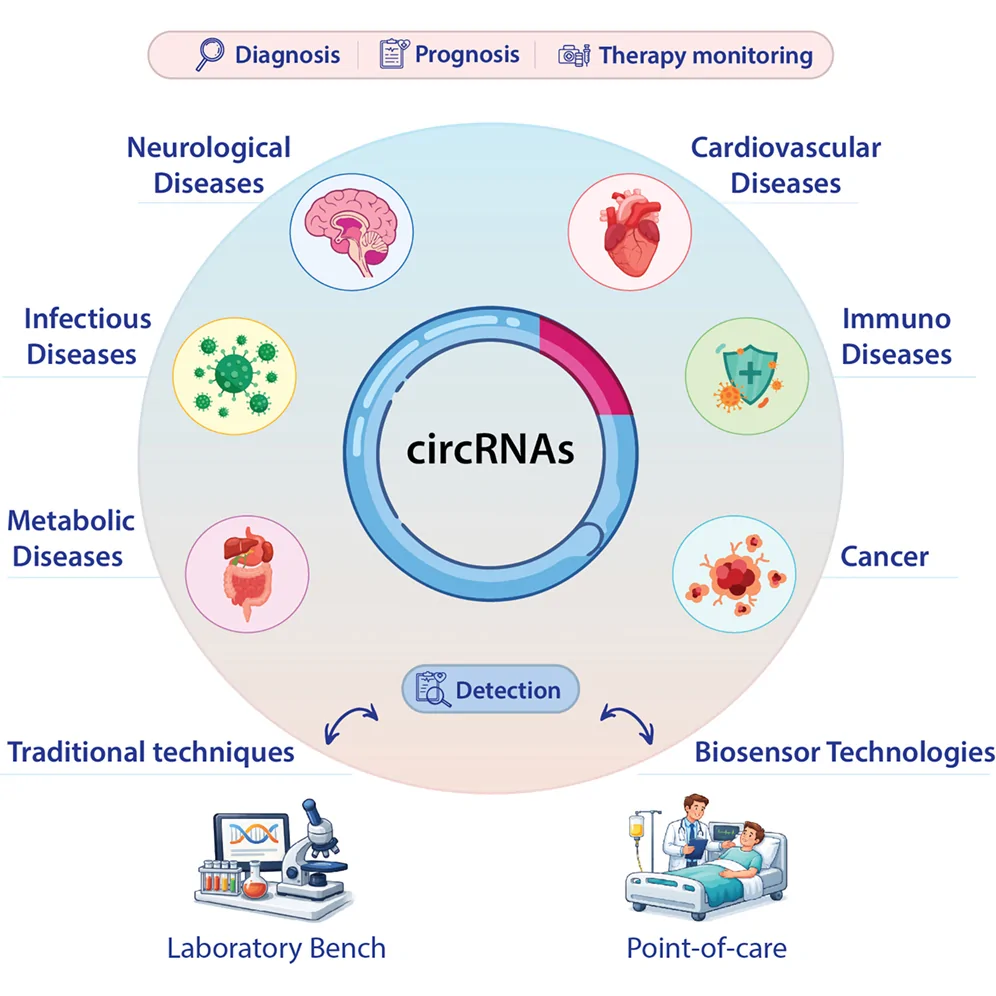

## Teaser

Diagnostic technique in health technology; Biomolecules; Properties of biomolecules

## Introduction

Circular RNAs (circRNAs) are single-stranded, covalently closed RNA molecules generated through back-splicing of pre-mRNA exons, in which a downstream splice donor joins an upstream acceptor.^1^ This process forms a continuous loop structure that is highly resistant to exonuclease-mediated degradation. Their biogenesis is regulated by *cis*-elements, such as intronic complementary repeats (e.g., Alu elements-short, repetitive sequences abundant in the human genome that can base-pair to bring splice sites into proximity), which facilitate RNA folding, and *trans*-acting factors including RNA-binding proteins (e.g., QKI, MBNL1, ADAR1) that compete with canonical splicing machinery. In addition, lariat intermediates produced during spliceosome activity can contribute to circRNA formation. circRNAs were discovered more than 40 years ago, but recent years have seen them rise to the forefront of RNA biology research due to their functional diversity and potential clinical relevance.^1^^,^^2^^,^^3^

CircRNAs are classified into endogenous and exogenous types. Endogenous circRNAs include exonic, exon-intron, and intronic circRNAs, while exogenous circRNAs in eukaryotes arise from back-splicing after mRNA cleavage and include satellite RNAs, virus-like RNA particles, and viroids. Some exogenous circRNAs require helper viruses for replication and movement, and a subset functions as non-coding RNAs.^2^ Functionally, circRNAs show highly specific, cell-type-dependent expression during development, suggesting tightly regulated biological roles. They participate in transcriptional and post-transcriptional regulation, acting as microRNA (miRNA) sponges, protein scaffolds modulating enzymatic activity, and interacting with RNA polymerase II to regulate transcription. In certain contexts, they also undergo cap-independent translation. Collectively, they influence miRNA regulation, protein synthesis, and broader gene regulatory networks.^1^^,^^2^^,^^3^

**Figure 1:**
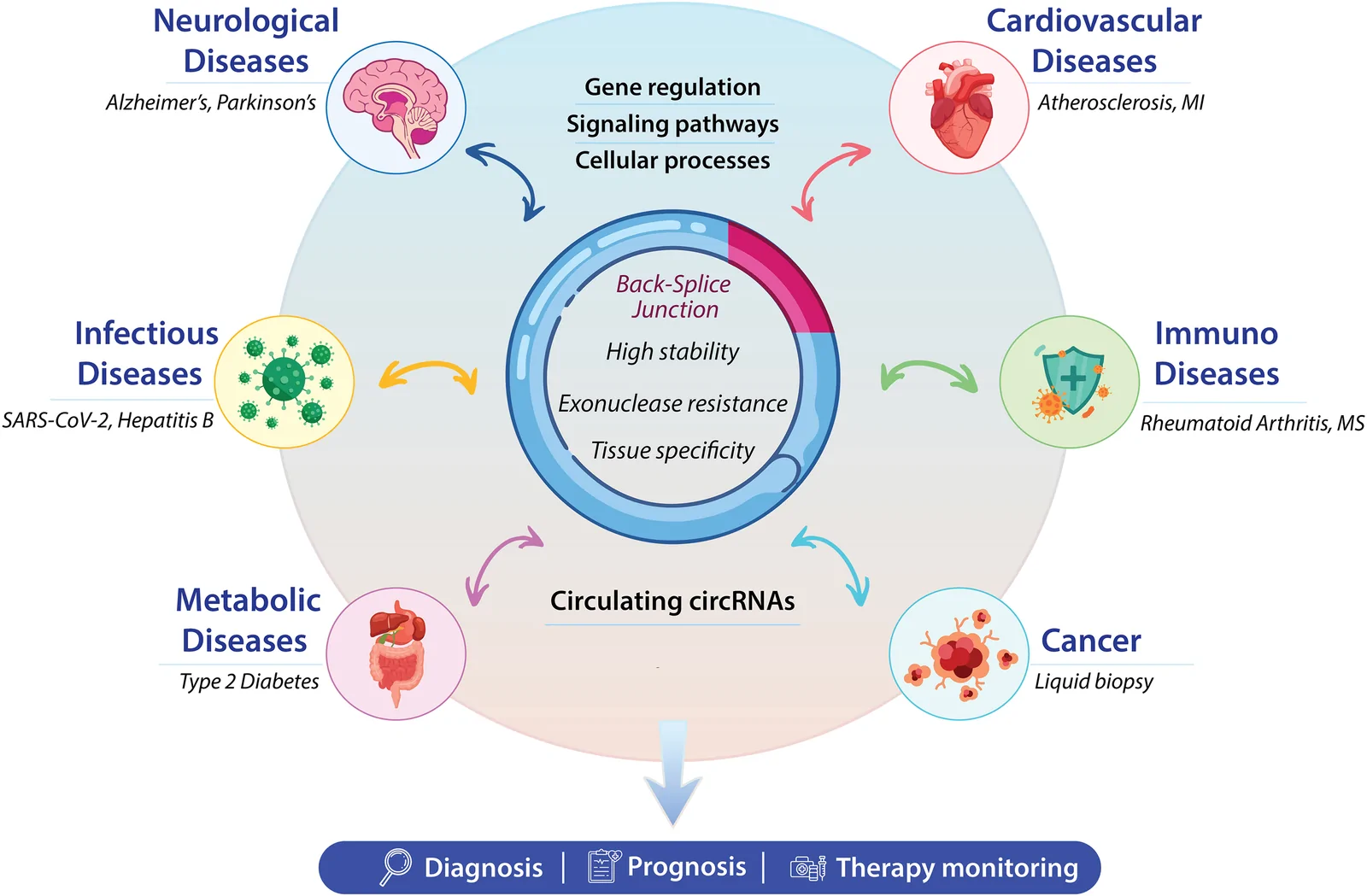
Schematic representation of circRNAs as diagnostic and prognostic biomarkers across human^9^ diseases The figure illustrates the key features of circRNAs, including the presence of a back-splice junction (BSJ), high stability, exonuclease resistance, and tissue specificity, as well as their involvement in gene regulation and cellular processes. circRNAs are associated with multiple disease categories, including neurological, cardiovascular, infectious, immune, metabolic diseases, and cancer. Circulating circRNAs detectable in biofluids highlight their potential as non-invasive biomarkers for diagnosis, prognosis, and therapy monitoring

circRNAs are particularly valuable as biomarkers due to their remarkable stability, cell-type-specific accumulation, and temporal regulation, despite the relatively low efficiency of back-splicing. They are enriched in non-proliferative cells such as neurons, where they increase during neurogenesis and localize at synapses, supporting development and maintenance, while their levels are often reduced in rapidly proliferating cells, such as cancer cells, but accumulate during aging. Their detectability in biofluids, including plasma and exosomes, makes them promising candidates for liquid biopsy applications, enabling non-invasive assessment of disease.^4^^,^^5^ This dynamic expression links circRNAs to a broad spectrum of human disorders, including organ-specific cancers, metabolic, cardiovascular, and neurodegenerative diseases, highlighting their potential for early detection, prognosis, therapy response, and deeper understanding of disease mechanisms.^6^^,^^7^ (Figure 1). However, practical application faces challenges, including low abundance, technical difficulties in accurately identifying back-splice junctions (BSJs), and sequencing artifacts. While approaches such as RNase R enrichment and long-read sequencing have improved detection, issues of standardization, reproducibility, and clinical validation continue to limit their widespread use.^8^

Thus, this perspective integrates current knowledge on circRNA biology, their roles in human disease, and the analytical strategies available for their detection. Because the clinical utility of circRNAs is inherently linked to the ability to detect them accurately, this review integrates advances in circRNA biomarker research with the analytical and biosensing technologies that support their translation into clinical practice.

### circRNAs in disease: Diagnostic and prognostic biomarkers

circRNAs have emerged as important diagnostic and prognostic biomarkers across a wide range of diseases.^9^ Although their association with pathological conditions spans multiple disease areas, the depth of available evidence varies substantially across them. A large proportion of current circRNA biomarker studies have focused on cancer, where larger patient cohorts, higher levels of validation, and a more extensive mechanistic understanding have been reported. Their relevance is particularly evident in triple-negative breast cancer, colorectal cancer, and gastric cancer, where altered circRNA expression is strongly associated with tumor development and progression. This emerging correlation is not limited to traditional invasive tissue biopsies but extends to the field of liquid biopsy, where circRNAs can be reliably detected in serum and plasma. In many cases, their dysregulation, most commonly upregulation, correlates with specific cancer types and clinical features.^10^^,^^11^

Beyond oncology, circRNAs also play a significant role in neurological and cardiovascular diseases, including Alzheimer’s disease, Parkinson’s disease, and stroke. In both cancer and these disorders, panels of circRNAs are increasingly being used to establish disease-specific signatures, enabling improved detection, patient stratification, and insights into underlying mechanisms and treatment responses.^12^^,^^13^ Additionally, circRNAs are gaining attention as biomarkers in autoimmune diseases, such as psoriasis, and in metabolic and inflammatory disorders, including Crohn’s disease. In these contexts, circRNAs not only support disease diagnosis but also provide valuable information on regulatory and mechanistic pathways.^9^^,^^14^ Finally, in the viral infections field, circRNAs can be dysregulated and directly correlated with the antiviral defense and the detection of the corresponding infections such as hepatitis B and C virus, H1N1 influenza virus, herpes simplex virus and so forth.^15^ While these applications are rapidly emerging, they generally remain supported by a more limited body of evidence compared to oncology.

To provide a practical overview, Table 1 summarizes representative examples, including specific circRNAs studied, cohort sizes, and whether clinical validation was performed. It also highlights the direction of circRNA dysregulation (up- or downregulation) and their correlation with disease states, offering a useful guide for both research and clinical applications.

**Table 1:** Representative diagnostic and prognostic circRNA biomarkers

Disease	circRNA	Cohort/study details	Key findings	Detection method	Reference
Early-stage NSCLC	circUSP10	40 patients vs. 40 controls (+10 paired tissues)	upregulated, correlates with tumor size/TNM; diagnostic AUC 0.896 (discovery)/0.829 (validation)	RT-qPCR	Bai et al.^16^
NSCLC	novel_circ_0005280	41 tumors vs. 27 adjacent normals	downregulated; linked to tumor diameter and age; low levels predict poorer prognosis.	RNA-seq/RT-qPCR validation	Li et al.^17^
TNBC	hsa_circ_0000479 (circEPSTI1)	38 paired TNBC tumors vs. normal; 240 TNBC pts (ISH on tissue microarray)	upregulated in >80% tumors; high levels linked to larger size, LN+, higher TNM, shorter DFS/OS; independent adverse prognostic marker	microarray/ISH validation	Chen et al.^18^
BC	hsa_circ_0104824	83 pts blood; 37 paired tissues; 49 healthy controls	downregulated in tissue/plasma; strong tissue-plasma correlation; good diagnostic potential	RT-qPCR	Li et al.^19^
BC (staging)	circMTO1, circCDYL, circHIPK3	13 tumors (8 I-II, 5 III) + 6 normals	Cas12a nanoswitch; downregulation of circMTO1 and upregulation of circCDYL and circHIPK3; discriminates stage I-II from stage III breast cancer (AUC = 1.0)	CRISPR-Cas12a nanoswitch assay	Liu et al.^20^
CRC	hsa_circ_0124554	40 paired CRC vs. adjacent tissues; 32 CRC plasma; controls	upregulated in CRC tissues/plasma; linked to differentiation, invasion; +CEA/CA19-9 improves Dx	qRT-PCR	Li et al.^21^
GC	8-circRNA panel hsa_circ_0001013 hsa_circ_0008768 hsa_circ_0034398 hsa_circ_0007380 hsa_circ_0052001 hsa_circ_0006089 hsa_circ_0002019 hsa_circ_0045602	194 GC vs. 94 controls (serum); matched tissues; post-op	panel AUC 0.87/0.83; detects early GC, distinguishes from other GI cancers; drops post-surgery	RT-qPCR	Roy et al.^22^
PDAC	hsa_circ_0060733, hsa_circ_0064288, hsa_circ_0006117, hsa_circ_0007895, hsa_circ_0007367	plasma samples from patients with PDAC and controls; two independent clinical cohorts	non-invasive early PDAC detection; high sensitivity/specificity vs. controls and benign conditions	RT-qPCR	Xu et al.^23^
Acute MI	MICRA	whole blood from 472 patients with acute MI assessed at reperfusion	MICRA independently stratified EF groups (adjusted OR 0.78 per log unit; 95% CI 0.64–0.95); improved multivariable model fit and risk stratification for post-MI LV dysfunction	RT-qPCR	Salgado-Somoza et al.^24^
AD	48 circRNAs	human hippocampus RNA-seq; neuronal precursor cells	48 circRNAs associated with AD; region/subtype-specific patterns; tau exposure downregulates similar circRNAs	RNA-seq/RT-qPCR validation	Puri et al.^25^
Early-stage PD	circRNAs from ESYT2, BMS1P1, CCDC9	PPMI: 259 PD/161 controls; ICICLE-PD: 48 PD/48 controls; blood RNA-seq	reduced circRNA expression in PD blood; limited diagnostic performance vs. linear RNA	RNA-seq	Whittle et al.^26^
AIS	circFUNDC1, circPDS5B, circCDC14A	discovery: 3 AIS/3 controls; Validation: 36/36; replication: 200/100 plasma	upregulated in AIS; panel AUC ≈0.875 (91% spec, 71.5% sens); change rates predict 7-day outcome	microarray/RT-qPCR validation	Zuo et al.^27^
PSCI	hsa_circ_0089762; hsa_circ_0064644; hsa_circ_0089763	China, LAA anterior circulation infarction; discovery: 4 PSCI/4 PSCN; validation: 45 PSCI/30 PSCN; plasma day 7	upregulated in PSCI; ROC AUC 0.993 (sensitivity 97.8%, specificity 96.7%); negatively correlates with MoCA (r = −0.693).	microarray/RT-qPCR validation	Chen et al.^28^
LN	circRNA_002453	two-stage: discovery microarray (5 L N, 5 SLE non-LN, 5 healthy); validation qRT-PCR (30 L N, 29 SLE non-LN, 26 RA, 27 healthy)	upregulated in LN vs. SLE non-LN/RA/controls; correlated with 24 h proteinuria (r = 0.57) and renal SLEDAI (r = 0.64); ROC AUC 0.91 (sens 0.90, spec 0.84) for LN detection	microarray/RT-qPCR validation	Ouyang et al.^29^
RA	hsa_circ_0001394; hsa_circ_0001998; hsa_circ_0009172; hsa_circ_0006732; circRNA2842; circRNA8330	two-phase PBMC study: discovery RNA-seq (3 treatment-naïve RA vs. 3 HCs); validation RT-qPCR (32 RA vs. 32 HCs)	six upregulated circRNAs differentiate in RA vs. HCs; strong diagnostic performance (AUC 0.99, specificity 100%, sensitivity >80%); positively correlated with RF (rs = 0.414, *p* = 0.018) and implicated in Wnt/calcium/VEGF pathways.	RNA-seq/RT-qPCR validation	Yang et al.^30^
T2DM	hsa_circ_0054633	three-step blood study: microarray (6 controls vs. 6 T2DM); 1st validation (20 controls, 20 prediabetes, 20 T2DM); large validation (60 controls, 63 pre-diabetes, 64 T2DM); qRT-PCR	demonstrates diagnostic capability for pre-diabetes and T2DM (AUCs up to 0.84)	microarray/RT-qPCR validation	Zhao et al.^31^
CD	hsa_circ_0062142	colon biopsies; microarray (4 CD, 4 UC, 4 HC); validation in additional CD/UC/HC biopsies	upregulated in CD vs. UC/HC (*p* < 0.01); AUC 0.803 for CD vs. non-CD, indicating favorable diagnostic value	microarray/RT-qPCR validation	Hu et al.^32^
CHB	circ_0004812	liver tissue biopsy and blood samples/25 patients vs. 25 healthy controls	upregulated in patients with CHB and cells;	qRT-PCR/western blotting	Zhang et al.^33^

**AD**: Alzheimer’s disease; **AIS**: acute ischemic stroke; **AUC**: area under curve; **BC**: breast cancer; **CA19-9**: carbohydrate antigen 19–9; **CEA**: carcinoembryonic antigen; **CD**: Crohn’s disease; **CHB**: chronic hepatitis B; **circCDYL**: chromodomain Y-like; circHIPK3: homeodomain-interacting protein kinase 3; circMTO1: circRNA mitochondrial transfer RNA translation optimization; **CRC**: colorectal cancer; **DFS**: disease-free survival; **DX**: improves diagnosis; **EF**: ejection fraction; **GC**: gastric cancer; **GI**: gastro-intestinal; **HC**: healthy control; **HCC**: hepatocellular carcinoma; **ISH**: *in situ* hybridization; **ICICLE-PD**: incidence of cognitive impairment in cohorts with longitudinal evaluation-PD; **LAA**: large artery atherosclerosis; **LN**: lupus nephritis; **LV**: left ventricular; **MI**: myocardial infarction; **MICRA**: myocardial infarction-associated circRNA; **MoCA**: Montreal Cognitive Assessment; **NSCLC**: non-small cell lung cancer; **OS**: overall survival; **PBMCs**: peripheral blood mononuclear cells; **PD**: Parkinson’s disease; **PDAC**: pancreatic ductal adenocarcinoma; **PPMI**: Parkinson’s progression markers initiative; **PSCI**: post-stroke cognitive impairment; **PSCN**: post-stroke cognitive normal; **qRT-PCR**: quantitative reverse transcription polymerase chain reaction; **RA**: rheumatoid arthritis; **RF**: rheumatoid factor; **rs**: risk stratification; **ROC**: receiver operating characteristic; **SLE**: systemic lupus erythematosus; **SLEDAI**: Systemic Lupus Erythematosus Disease Activity Index; **TNBC**: triple-negative breast cancer; **TNM**: tumor-node-metastasis; **T2DM**: type 2 diabetes mellitus; **UC/HC**: ulcerative colitis/healthy control; **VEGF**: vascular endothelial growth factor.

Although numerous circRNAs have demonstrated diagnostic and prognostic potential across a broad range of diseases (Table 1), their practical implementation as biomarkers remains strongly dependent on reliable detection technologies. Therefore, understanding the strengths and limitations of currently available analytical methods is essential for assessing the translational potential of circRNA-based diagnostics and for selecting the most appropriate strategy for specific clinical and research applications.

### Traditional analytical strategies for circRNA detection

CircRNA analysis presents specific analytical challenges mainly related to their circular structure and generally low abundance in biological matrices. Due to their lack of 5′-3′ polarity and poly(A) tails, circRNAs are depleted during conventional mRNA enrichment procedures, requiring total RNA or rRNA-depleted RNA as input material.^34^ Consequently, circRNA profiling has primarily relied on high-throughput approaches such as RNA sequencing (RNA-seq) and circRNA-specific microarrays.^35^ In RNA-seq-based workflows, circRNA detection relies on sequencing reads spanning the BSJ, which represents the sequence feature distinguishing circular transcripts from linear RNAs.^36^^,^^37^ Computational pipelines detect BSJ reads using annotation-dependent or independent strategies; however, library artifacts and biological events like *trans*-splicing can cause false positives, making validation with complementary techniques essential, particularly for novel circRNA candidates. The low abundance of many circRNAs limits the reliability of RNA-seq-based quantification, especially in small or highly diluted samples, and complicates comparative analyses due to low signal-to-noise ratios and sample heterogeneity. Consequently, accurate quantification of less abundant circRNAs requires deeper sequencing and longer read lengths (>100 nucleotides).^36^ Overall, circRNA detection currently relies on a range of analytical strategies that differ substantially in terms of sensitivity, throughput, experimental complexity, and suitability for low-abundance targets. These approaches include sequencing-based methods, hybridization assays, and amplification-based techniques, each offering distinct advantages while also presenting inherent limitations that constrain their broader analytical and clinical applicability (summarized in Table 2).

**Table 2:** Summary of commonly used circRNA detection methods, including their key features, strengths, and limitations for analytical and clinical applications

Technique	Cost	Experimental setup	Pros	Cons	Reference
RNase R-assisted RT-qPCR	$–$$	RNA extraction; RNase R digestion; RT; qPCR (BSJ- primers)	high sensitivity; widely accessible	incomplete RNase R digestion; RT/primer bias; low throughput	Wang et al.^38^
uMRT-based RT-PCR	$$	RNA extraction; uMRT-based RT; PCR (BSJ primers)	improved RT efficiency; higher reproducibility	enzyme-dependent; requires thermal cycling	Warkentin et al.^39^
RT-ddPCR	$$$	Plasma RNA; RT; droplet generation; PCR; fluorescence readout	absolute quantification; very high sensitivity	costly instrumentation; multistep workflow; limited portability	Ji et al.^40^
ddPCR for circRNA validation	$$$	RNA extraction; RT; droplet generation; PCR; readout	high quantitative accuracy; ideal for validation	not high-throughput; specialized instrumentation	Li et al.^41^
RT-RCA	$–$$	RNA extraction; RT; isothermal RCA; fluorescence readout	high circRNA selectivity; rapid; isothermal	enzyme-dependent; limited multiplexing: single-target assay	Liu et al.^42^
RCA-based nucleic acid sensing assay	$–$$	circular template recognition; primer binding; RCA; fluorescence readout	strong signal amplification; isothermal	not circRNA specific; enzyme-dependent	Boss et al.^43^
Multiple stem-loop primer cascaded LAMP	$$	RNA extraction; multiple stem-loop primers; LAMP; fluorescence readout	discriminates circ/linear RNA; rapid	complex primer design; enzyme-dependent	Liu et al.^44^
Traditional Northern blot for circRNA validation	$$	RNA extraction; denaturing gel electrophoresis to remove secondary structure; transfer to membrane; probe hybridization	direct evidence of size and circular topology	labor-intensive; large RNA input	Schneider et al.^45^
Non-radioactive Northern blot with DIG-labeled probes	$$	RNA extraction; gel electrophoresis; membrane transfer; hybridization with DIG-labeled probe; chemiluminescent detection	avoids radioactivity; direct size and circularity validation	high RNA input; time-consuming	Wang et al.^46^
circFISH for concurrent imaging of linear and circRNAs	$$	fixed cells/tissue; BSJ and linear probes; RNase R pretreatment; high-resolution fluorescence imaging	simultaneous circ/linear detection	dual probe complexity; long protocol	Koppula et al.^47^
Microarray	$$	RNA extraction; RNase R treatment; circRNA microarray hybridization; fluorescence scanning	high-throughput detection; identification of clinically relevant circRNAs; applicable to human tumor tissues.	platform-limited discovery	Zhang et al.^48^
Large-scale RNA-seq circRNA detection with CIRI3 bioinformatic pipeline	$$	large RNA-seq datasets; BSJ detection	scalable to very large datasets; high accuracy	deep sequencing required; heavy computation	Zheng et al.^8^
Stem-loop primer-induced LAMP	$$	RNA extraction; stem-loop primers; LAMP; fluorescence readout	ultra-sensitive; fast; no thermal cycling	complex primer design: multiple primers set	Zhang et al.^49^
Amplified FISH (ampFISH)	$$	fixed cells; BSJ probes; HCR amplification; fluorescence imaging	**s**ingle-molecule sensitivity; *in situ* detection	advanced microscopy; complex labeled probes	Jaijyan et al.^50^

$: clinical routine cost; $$: moderate cost; $$$: high cost; **RT**: reverse transcription; **PCR**: polymerase chain reaction; **qPCR**: quantitative polymerase chain reaction; **uMRT**: UltraMarathonRT; **ddPCR**: digital droplet PCR; **FISH**: fluorescence *in situ* hybridization; **RCA**: isothermal rolling circle amplification; **LAMP**: loop-mediated isothermal amplification; **BSJ**: back-splice junction; **HCR**: hybridization chain reaction.

### Amplification-based methods

Amplification-based approaches represent the most widely used strategies for circRNA detection, owing to their high sensitivity and relative accessibility. These methods typically rely on divergent primers targeting the BSJ and include quantitative reverse transcription PCR (RT-PCR), digital PCR, and various isothermal amplification schemes.

### PCR-based detection

RT-PCR and quantitative RT-PCR (RT-qPCR) are widely used amplification-based methods for circRNA detection and are routinely employed to validate circRNAs identified by RNA-seq.^51^ These approaches rely on divergent primers spanning the BSJ and offer high analytical sensitivity, broad dynamic range, and straightforward experimental workflows, making them well suited for routine laboratory use.^35^^,^^52^ Despite these advantages, several intrinsic limitations affect the quantitative accuracy of RT-PCR-based circRNA analysis. Incomplete digestion of linear RNAs by RNase R, particularly for transcripts containing stable secondary structures at the 3′ end, can lead to false-positive amplification signals.^53^ In addition, the circular structure of circRNAs allows reverse transcriptase to generate rolling-circle-like cDNA products, producing repeated sequences that can lead to systematic overestimation of circRNA levels during PCR amplification.^54^ Primer design further represents a critical step, as poorly designed primers may fail to discriminate circRNAs from homologous linear mRNAs or distinct circRNA isoforms sharing the same BSJ.

Representative PCR-based workflows combining RNase R treatment and RT-qPCR have been used to mine, validate, and quantify circRNA transcriptomes.^38^ Recent improvements include the optimization of the reverse transcription step. For example, highly processive reverse transcriptases, such as UltraMarathonRT (uMRT),^39^ enhance continuous cDNA synthesis and reproducibility. Ligation-assisted PCR assays such as SplintQuant, selectively ligate probes at the BSJ, enabling specific amplification without reverse transcription. Such assays achieve limits of quantification down to the femtomolar range and exhibit high reproducibility and cost-effectiveness; however, they still rely on thermal cycling and laboratory-based instrumentation, limiting their portability and suitability for point-of-care applications.

Alternative PCR-based strategies include digital droplet PCR (ddPCR), which enables absolute circRNA quantification by partitioning reactions into thousands of droplets and applying Poisson statistics, reducing amplification bias and eliminating external calibration.^55^ ddPCR has been also applied to plasma-based assays, such as the detection of circHIPK3 in patients with hepatocellular carcinoma (HCC)^40^ and as an orthogonal tool to validate RNA-seq-based circRNA quantification, including allele-specific expression and circularization efficiency.^41^ While highly sensitive, RT-ddPCR requires specialized and costly instruments.^51^^,^^56^

### Isothermal amplification

Rolling circle amplification (RCA) is an isothermal technique that amplifies circular templates into long single-stranded products, making covalently closed circRNAs ideal substrates for sensitive and efficient circRNA detection.^57^ Representative applications of RCA for circRNA detection have been reported in solution-phase assays. These approaches exploit the circular structure of circRNAs to enable selective isothermal amplification coupled with fluorescence-based readouts, supporting their applicability for circRNA analysis in complex biological samples. Related RCA-based strategies generate long single-stranded amplification products that facilitate sensitive fluorescence detection, further highlighting the adaptability of RCA for nucleic acid sensing applications.^42^^,^^43^ Beyond RCA, other isothermal amplification strategies have also been explored for circRNA detection, aiming to achieve rapid signal amplification under constant temperature conditions without thermal cycling. One prominent example is loop-mediated isothermal amplification (LAMP), which uses strand-displacing DNA polymerases, such as Bst polymerase, together with multiple primer sets (typically four to six primers) targeting distinct regions of the target sequence to drive efficient amplification.^58^ When adapted for circRNA analysis, LAMP assays use stem-loop primer designs that span the BSJ. These primers form secondary structures that initiate amplification only in the presence of the circular junction, allowing selective discrimination of circRNAs from linear transcripts without RNase R pretreatment. The incorporation of additional stem-loop primers can further accelerate the reaction, enabling circRNA detection within short assay times (≤35 min) and achieving femtomolar sensitivity.^44^ Compared with conventional PCR-based approaches, LAMP generally offers similar or higher sensitivity while requiring lower instrumentation complexity, reduced reagent consumption, and lower operational costs.^58^

Despite these advantages, LAMP-based circRNA detection faces notable analytical challenges. Primer design is inherently complex, as multiple primers must be optimized for BSJ specificity, while the strong strand-displacement activity of Bst polymerase can cause nonspecific amplification and background signals under isothermal conditions.^59^ Additionally, advanced stem-loop-based LAMP variants achieve attomolar sensitivity, yet the resulting assay complexity and limited robustness continue to constrain their broader clinical and analytical application.^58^

### Hybridization-based methods

Hybridization-based methods detect circRNAs through sequence-specific base pairing with complementary probes, often spanning the BSJ to distinguish circular from linear transcripts. They generally offer simpler workflows and lower enzymatic bias than amplification-based approaches, though sensitivity is reduced. In some cases, hybridization assays can also be applied to pre-amplified targets to enhance detection.

### Northern blotting

Northern blotting is a probe-based technique that detects specific RNA species after electrophoretic separation and transfer onto a solid membrane. Because it does not involve reverse transcription or enzymatic amplification, it avoids amplification-related bias and is widely regarded as the reference method for confirming circRNA presence, size, and circular topology. However, its practical application is limited by the need for relatively large RNA inputs, labor-intensive procedures, low sensitivity, and limited throughput, making it unsuitable for low-abundance or large-scale circRNA profiling.^60^

Enrichment strategies using RNase R can also introduce ambiguities, as structured linear RNAs may resist digestion while large circRNAs may be partially degraded, affecting result interpretation.^45^ Despite these challenges, Northern blotting uniquely allows direct assessment of RNA size and circular configuration, features not readily accessible with amplification-based assays.^35^ To improve usability, technical refinements have been proposed: Non-radioactive approaches using digoxigenin (DIG)-labeled probes enhance safety while preserving specificity and size resolution,^46^ and protocols combining RNase R and RNase H treatments improve discrimination between circular and linear RNAs.

### Fluorescence *in situ* hybridization (FISH)

Fluorescence in situ hybridization (FISH), a technique traditionally used to detect DNA or linear RNA, has been specifically adapted for circRNAs, enabling their visualization in fixed cells and tissues with probes targeting the BSJ to distinguish circular from linear transcripts. However, high sequence overlap with linear RNAs can cause off-target hybridization, often requiring RNase R pretreatment.^61^

Several adaptations improve circRNA specificity and sensitivity. Circular FISH (circFISH) uses dual-color probe sets to independently label circular and linear transcripts, allowing identification via co-localized fluorescence at single-molecule resolution.^47^ Building on this concept, BaseScope employs paired “Z probes” that generate amplified signals only when bound to adjacent sequences, reducing linear RNA interference.^62^ Amplified FISH (ampFISH) extends these strategies by coupling BSJ recognition with hybridization chain reaction (HCR) to detect low-abundance circRNAs *in situ*.^63^ More advanced ampFISH variants integrate hairpin-probe-initiated HCR with single-molecule FISH, enabling selective quantification of circular and linear transcripts within individual cells, with specificity confirmed by RNase R treatment.

Despite these advances, FISH-based circRNA assays remain limited by labor-intensive workflows, complex probe design, and reliance on high-resolution microscopy, constraining throughput and scalability for routine analytical or clinical applications.^64^

## Microarrays

CircRNA microarrays are high-throughput hybridization platforms that employ probes targeting BSJ sequences to selectively profile circRNAs. Owing to their scalability, these assays have been widely used to compare circRNA expression patterns between diseased and normal tissues and to identify dysregulated circRNAs with potential clinical relevance. CircRNA candidates identified by microarray analysis are commonly validated by RT-qPCR or RNA-seq, confirming their utility as screening tools.^65^ CircRNA microarray workflows are similar to conventional mRNA microarrays, with specificity achieved through BSJ-targeting probes. Total RNA is typically treated with RNase R to remove linear transcripts prior to labeling and hybridization on commercial circRNA arrays, such as those provided by Arraystar, CapitalBio Technology, and CD microarrays, which incorporate external RNA controls to improve data reliability. Li et al. applied an Arraystar circRNA microarray to profile differential circRNA expression in endometrial carcinoma tissues and validated selected candidates by RT-qPCR, illustrating the utility of microarrays for large-scale circRNA screening in clinical samples.^66^ Zhang et al. applied a circRNA microarray to profile differential expression of circRNAs in colorectal cancer tissues compared with adjacent non-tumor controls, identifying dysregulated circRNAs associated with metastasis and prognosis.^48^ Despite their high detection efficiency and suitability for large-scale circRNA screening, circRNA microarrays require substantial RNA input, provide only relative quantification, and preclude direct assessment of circRNA-to-linear RNA ratios due to RNase R pretreatment. As a result, their application is largely confined to discovery studies rather than quantitative or diagnostic circRNA analysis.^64^

### Sequencing-based methods

RNA-seq has been central to the genome-wide discovery and annotation of circRNAs. Short-read RNA-seq detects circRNAs by identifying reads that span the BSJ, typically using libraries depleted of rRNA or treated with RNase R to enrich circRNAs. However, because reads are relatively short, this approach often cannot reconstruct the full-length sequence of circRNAs or resolve complex alternative splicing events within them.^67^^,^^68^ Low-abundance circRNAs are often missed at standard sequencing depths, a limitation that can be addressed by long-read sequencing. Platforms such as nanopore-based technologies directly sequence full-length circRNAs, enabling accurate identification of internal exon composition and alternative splicing events.^69^^,^^70^ Strategies such as long-read nanopore sequencing of nicked circRNAs (circNick-LRS) and targeted long-read panels have substantially expanded the catalog of circRNAs in mammalian tissues.^71^

Nevertheless, long-read sequencing requires more demanding library preparation, exhibits higher error rates, and incurs greater costs compared with short-read approaches.^68^ Computational pipelines, such as the CIRI3 tool, enhance circRNA detection from large RNA-seq datasets by efficiently identifying BSJ reads, enabling analysis of thousands of cancer samples and construction of circRNA biomarker networks.^8^ Despite their unparalleled ability for unbiased discovery and structural characterization, sequencing-based methods rely on complex bioinformatic workflows and typically require orthogonal validation, limiting their suitability for routine quantification or clinical use.^35^

Collectively, current circRNA detection methods offer several strengths but also share important limitations. Amplification-based approaches (e.g., RT-qPCR, ddPCR, RCA/LAMP) provide high sensitivity, accurate quantification, and broad accessibility, making them widely used for targeted validation. Hybridization-based methods (e.g., Northern blot, FISH, microarrays) enable specific BSJ detection and valuable spatial or visual localization within cells and tissues. Sequencing-based techniques (short- and long-read RNA-seq) allow comprehensive transcriptome-wide analysis, structural characterization, and discovery of novel circRNAs.

**Figure 2:**
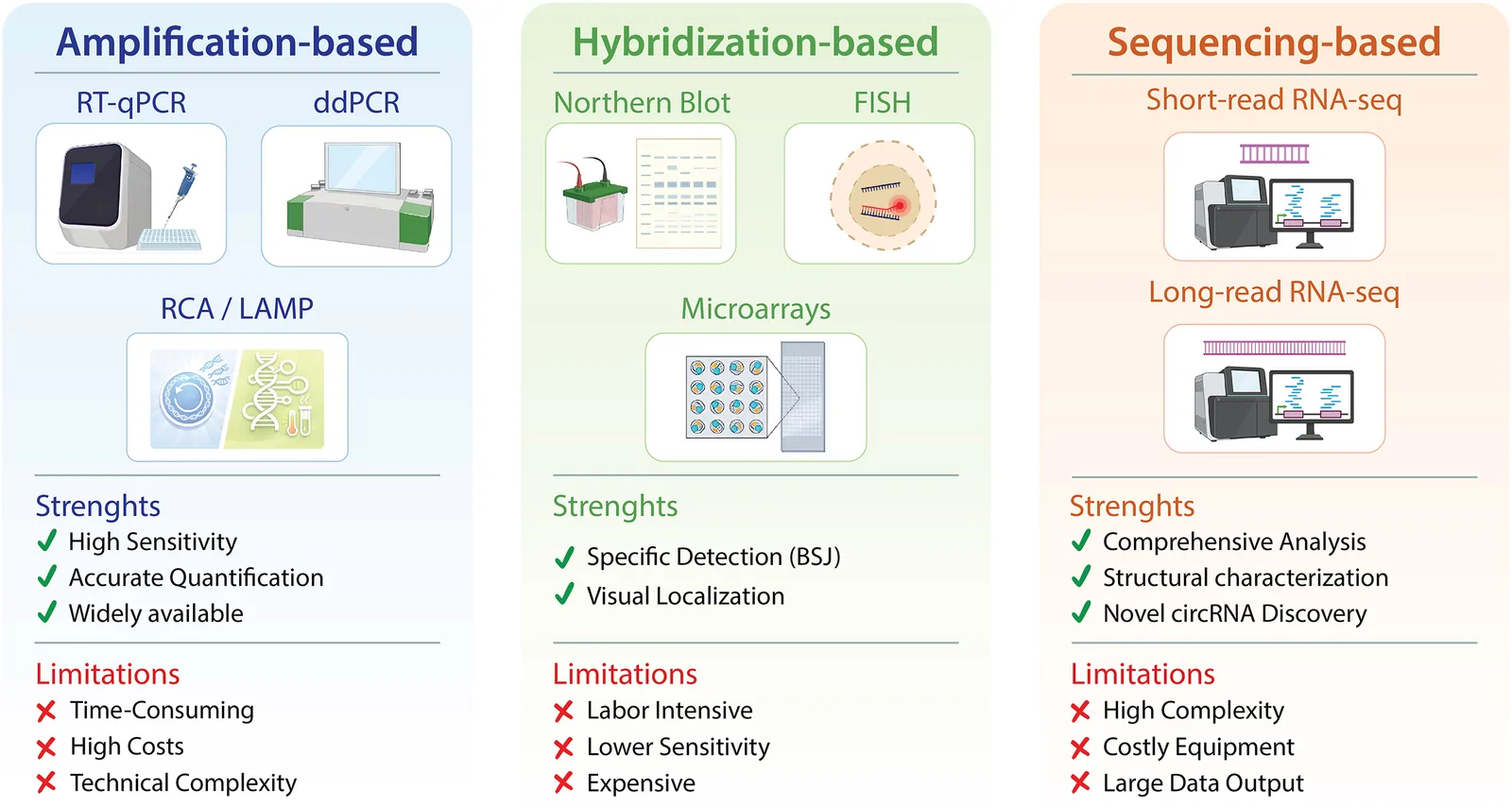
Schematic representation of traditional analytical strategies for circRNA detection The figure summarizes conventional methods for circRNA detection, categorized into amplification-based, hybridization-based, and sequencing-based approaches. Amplification-based techniques include RT-qPCR, droplet digital PCR (ddPCR), and isothermal amplification methods (RCA/LAMP). Hybridization-based methods comprise Northern blotting, fluorescence *in situ* hybridization (FISH), and microarrays, enabling specific detection and localization. Sequencing-based approaches, including short- and long-read RNA sequencing, allow comprehensive circRNA profiling. Key strengths and limitations of each strategy are highlighted

Despite these advantages, significant challenges remain. Amplification-based methods can be time-consuming, costly, and prone to amplification bias, particularly when enzymatic steps such as RNase R treatment are required. Hybridization-based techniques are often labor-intensive, less sensitive for low-abundance circRNAs, and relatively expensive. Sequencing approaches, while powerful, involve high technical complexity, costly instrumentation, and generate large datasets requiring advanced computational analysis. Across all methods, issues such as limited sensitivity for low-copy targets, workflow complexity, reduced scalability, and variability in reproducibility can constrain quantitative accuracy and clinical applicability. (Figure 2).

An additional challenge in circRNA biomarker studies is the substantial pre-analytical and analytical variability across experimental workflows. Factors such as sample source (e.g., tissue, plasma, serum, exosomes, or whole blood) can influence circRNA abundance and composition, while differences in RNA extraction and preparation procedures may affect RNA yield and integrity. Variability in RNase R enrichment efficiency can alter the proportion of circular versus linear transcripts detected, whereas library preparation protocols, sequencing depth, and normalization strategies can significantly impact circRNA quantification and the identification of low-abundance targets.^36^^,^^37^^,^^51^

Furthermore, different analytical platforms, including RNA-seq, microarrays, RT-qPCR, and emerging biosensor-based approaches, exhibit distinct sensitivity, specificity, dynamic ranges, and data-processing requirements, which can complicate direct comparisons across studies.^35^^,^^51^ As a result, reproducibility and cross-study validation remain challenging, highlighting the importance of standardized workflows and reporting practices to facilitate clinical translation.

From a practical perspective, the choice of the analytical method should be guided by the intended application. Sequencing-based approaches remain the preferred option for circRNA discovery and transcriptome-wide profiling, owing to their ability to identify novel circRNAs and characterize their structure. For targeted validation and quantitative analysis of previously identified candidates, RT-qPCR and ddPCR provide the most accessible and reliable solutions, with ddPCR offering the additional advantage of absolute quantification. Hybridization-based methods such as FISH are particularly valuable when spatial localization and cellular distribution need to be investigated, whereas Northern blotting remains an important orthogonal validation tool for confirming circRNA size and circularity. In liquid biopsy applications, RT-qPCR and ddPCR are currently the most established approaches due to their sensitivity and compatibility with low-abundance circulating targets. By contrast, emerging biosensor technologies are particularly attractive for near-patient testing and future point-of-care applications, as they combine rapid analysis, high sensitivity, simplified workflows, and the potential for miniaturization and portable operation. In this context, emerging biosensor technologies provide promising solutions by enabling rapid, highly sensitive, and potentially amplification-free detection.^72^^,^^73^^,^^74^ Although their clinical translation still requires further validation and standardization, these approaches simplify workflows, reduce reliance on complex instrumentation, and improve performance in complex biological samples, thereby enhancing the feasibility of robust circRNA detection strategies. These advances and their practical applications are discussed in the following section.

## Emerging biosensor technologies for circRNA detection

Biosensors for circRNA detection are generally built on two fundamental components: a molecular recognition layer that selectively binds the target RNA and a signal transducer that converts the binding event into a measurable output. For circRNA targets, selectivity is most commonly achieved by exploiting the BSJ as a unique molecular signature, using immobilized nucleic acid probes or structurally engineered analogs that remain stable in complex biological environments.^75^^,^^76^^,^^77^ This recognition strategy mirrors general nucleic-acid biosensing principles, where hybridization-driven specificity is combined with surface-confined probe architectures to maximize signal-to-noise ratios.^78^ Signal transduction in circRNA biosensors follows established biosensing paradigms and is typically achieved through electrochemical or optical mechanisms. Electrochemical biosensors translate target binding into changes in current, potential, or impedance, often enhanced by nanostructured interfaces that increase surface area and probe density, enabling sensitive detection with compact and low-power instrumentation. Optical biosensors rely on the modulation of fluorescence, plasmonic responses, or Raman scattering signals induced by circRNA-probe interactions on nanostructured substrates, allowing label-free or minimally labeled detection with high analytical sensitivity.^79^ A further general principle is the integration of biosensing elements with miniaturized and automated platforms, such as microfluidic or paper-based devices. These systems combine molecular recognition, fluid handling, and signal readout within a single architecture, supporting low sample consumption, multiplexing, and portability, key requirements for point-of-care applications.^80^^,^^81^^,^^82^ Together, these assays’ principles illustrate how circRNA biosensors leverage established biosensing concepts to enable rapid, sensitive, and potentially amplification-free detection in formats that are difficult to achieve with conventional laboratory-based assays.

## Scope and current limitations of biosensor concepts

Despite substantial progress in biosensor design and performance, the current landscape of biosensor technologies for nucleic acid detection still faces analytical and translational constraints. A recurring challenge is the performance gap between controlled test conditions and complex biological matrices; nonspecific interactions, background interferents, and variability in sample composition can reduce sensitivity and reproducibility when moving from buffers to real clinical samples.^83^ Another limitation relates to standardization and robust manufacturing. Many high-sensitivity biosensors rely on nanostructured surfaces, complex probe immobilization, or finely tuned microfluidic architectures, which can introduce batch-to-batch variability and complicate reproducibility across different production lots and laboratories. This complicates efforts to scale up fabrication and achieve consistent results in routine use.^76^ Nevertheless, biosensors retain intrinsic advantages such as rapid response, high analytical sensitivity, compatibility with miniaturized platforms, and suitability for portable analytical formats. Continued improvements in probe design, surface chemistry, and microfluidics are progressively addressing existing drawbacks, suggesting that the integration of biosensors into clinically relevant circRNA detection workflows is increasingly feasible.

Against this background, recent biosensor approaches for circRNA detection can be viewed as practical implementations of these general principles, aiming to address key analytical challenges while preserving the intrinsic advantages of biosensing technologies.

## Categories of circRNAs biosensors

In hybridization-based configurations, circRNA binding, mediated by BSJ-specific DNA or peptide nucleic acid (PNA) probes, results in a measurable change in the electrochemical signal depending on the sensing design. In signal-off systems, this interaction leads to a decrease in current due to steric hindrance, probe displacement, or impaired interfacial electron transfer. Conversely, signal-on configurations generate an increased electrochemical response by enhancing charge transfer or by increasing the density of electroactive species at the electrode surface. A representative example of the signal-off strategy is the point-of-care testing (POCT) assay for the detection of circ-CDYL in HCC, reported by Zhang et al.^76^ The sensing interface consists of a carbon fiber microelectrode modified with gold nanoflowers, which provide a suitable surface for the immobilization of thiolated PNA probes through Au–S bonds. Upon specific target binding and in the presence of an added redox mediator, interfacial electron transfer is hindered, resulting in a decrease in the measured electrochemical current. The sensor achieved quantitative detection over a wide concentration range (10 fM–1 μM), with a limit of detection (LOD) of 3.29 fM. Its high specificity, confirmed by single-base mismatch discrimination, was further demonstrated in human serum samples, highlighting its potential for clinical applications in early-stage HCC diagnosis.

**Figure 3:**
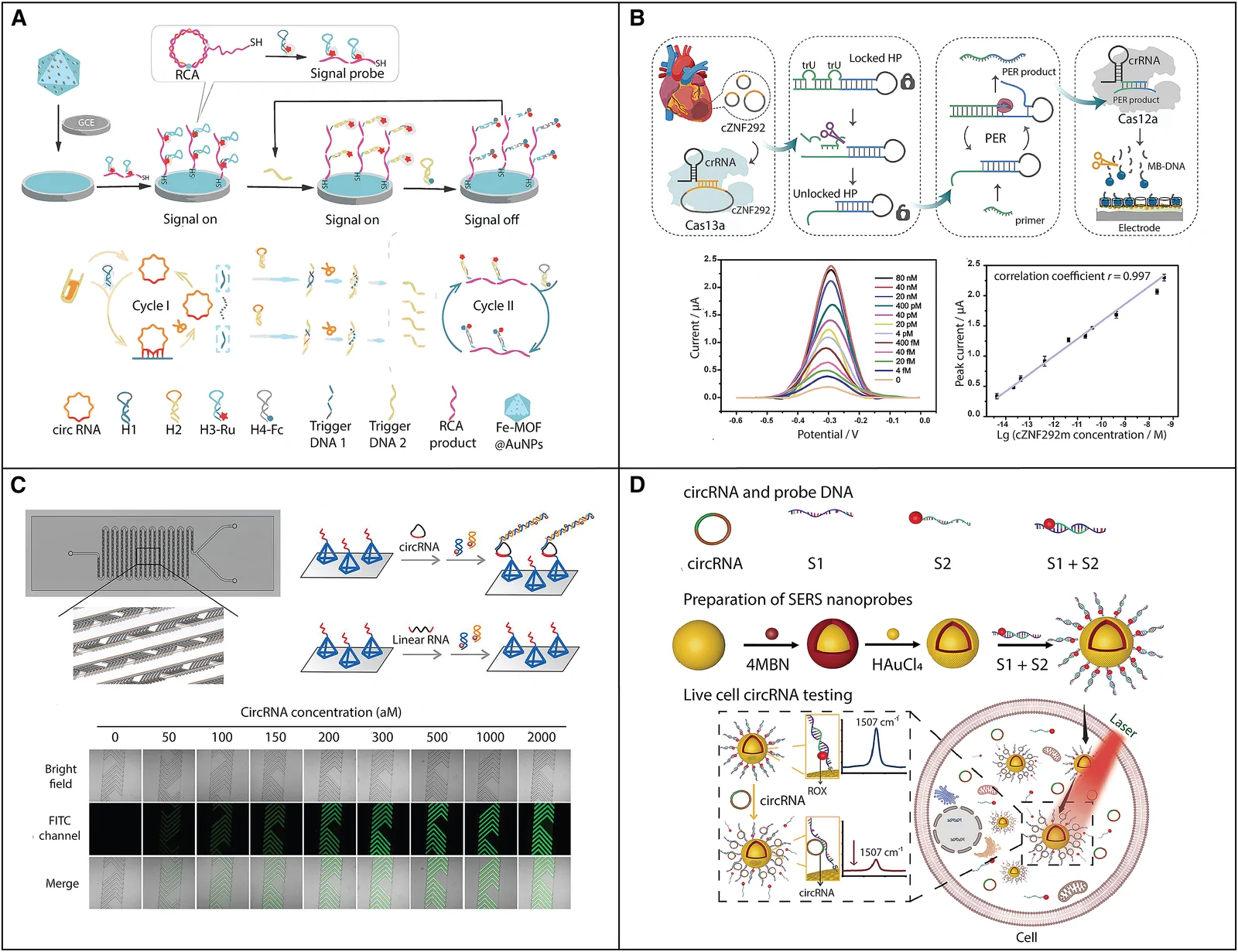
Representative examples of emerging biosensor technologies for circRNA detection The figure presents selected examples of advanced biosensing strategies for circRNA detection. (A) Electrochemiluminescence biosensor based on nanomaterial-enhanced signal amplification and rolling circle amplification for ultrasensitive detection of circRNA.^81^ (B) Electrochemical biosensor integrating a collaborative CRISPR-Cas system for highly specific detection of circulating circRNA via back-splice junction recognition.^83^ (C) Microfluidic biosensor functionalized with tetrahedral DNA nanostructures enabling selective capture and amplification-based detection of circRNA in complex samples.^84^ (D) Optical biosensor based on surface-enhanced Raman spectroscopy (SERS) for sensitive detection and imaging of circRNA in living cells.^85^ These approaches highlight recent advances toward sensitive, specific, and potentially point-of-care circRNA detection

Expanding this approach, capture probe-based hybridization strategies combined with target amplification methods have enabled the detection of circRNAs at clinically relevant concentrations, achieving sensitivities in the sub-femtomolar range. Building on these combined strategies, Zhang et al.^84^ developed an electrochemiluminescence (ECL)-based platform for circRNA detection, integrating a triple-amplification strategy (Figure 3A). The system is based on a Fe-MOF@AuNP-modified electrode, where Fe-based metal-organic frameworks functionalized with gold nanoparticles provide a conductive, high-surface-area interface for efficient probe immobilization and signal transduction. RCA, together with cyclic amplification processes, enables target-triggered signal amplification. The platform achieved a low LOD, down to 0.046 fM under optimized experimental conditions and showed preliminary ability to discriminate between patients with bladder cancer and healthy controls through direct, non-invasive circRNA detection in urine samples.

While these hybridization-driven platforms offer high sequence specificity, their analytical performance is often limited by modest signal amplification and reduced sensitivity at ultra-low target concentrations. To overcome these limitations, CRISPR/Cas-based systems have been increasingly integrated as a secondary enzymatic amplification layer, transforming hybridization events into highly amplified electrochemical outputs through collateral cleavage activity. When integrated with voltametric readouts, they allow fast signal generation and are well suited for rapid testing formats. Cheng et al.^85^ designed a Cas13a-driven electrochemical platform for the analysis of bladder cancer-associated circRNA directly in urine samples. In this configuration, DNA tetrahedron nanostructures are immobilized at the electrode interface and serve as a scaffold for methylene blue (MB), which acts as the electroactive reporter. Upon the recognition of the target circRNA, Cas13a is activated and cleaves uracil-containing sites within the tetrahedral structure, leading to its structural disruption and the release of the redox mediator from the electrode surface. This process results in a measurable decrease in the electrochemical signal. The assay enables, within 10 min, direct analysis of circRNA in small-volume urine samples, supporting its applicability for rapid and minimally invasive bladder cancer detection. To further enhance sensitivity, more advanced architectures have been developed by coupling CRISPR-based recognition with cascade amplification mechanisms. In this context, Sha et al.^86^ developed a collaborative CRISPR-Cas electrochemical platform that integrates the circRNA-targeting capability of Cas13a with cascade *trans*-cleavage activities of multiple Cas effectors, using a primer exchange reaction (PER) as an isothermal amplification bridge. Upon recognition of the BSJ, Cas13a activation initiates the PER process, generating intermediates that subsequently activate Cas12a and trigger amplified *trans*-cleavage of MB-labeled DNA reporters. This cascade mechanism produces a significantly enhanced electrochemical signal, enabling reliable detection of circulating circRNA directly in whole blood samples under the tested conditions. The system achieves excellent sensitivity, with a LOD of 2.13 fM in buffer, supporting its proof-of-concept applicability for high-sensitivity acute myocardial infarction (AMI) diagnosis (Figure 3B).

In the context of cyclic signal amplification strategies, recent efforts have focused on designing multi-layered nucleic acid amplification architectures that enhance both signal generation and target selectivity. In this context, Dong et al.^86^ developed a signal-on fluorescence platform for the detection of low-abundance circRNAs in biological matrices. In this system, RT-RCA generates long DNA strands containing repeated sequences that trigger a two-dimensional HCR network, leading to stable DNA nanostructures that activate FRET-based hairpin probes. This architecture achieves sub-picomolar sensitivity while improving discrimination against linear RNA isoforms without the need for additional enrichment or pretreatment steps.

The method demonstrated reliable performance in human tumor cell lines and tissue samples, highlighting its potential as a simple and robust platform for circRNA analysis in cancer-related experimental settings. Continuing in the field of fluorescent-based approaches, Liu et al.^79^ achieved attomolar circRNA detection in cancer tissues using a programmable quantum dot (QD)-based nanosensor. In this system, target recognition drives the formation of a stable three-way junction (TWJ) scaffold, which triggers cascade amplification and the generation of multiple linker probes that assemble into QD-DNA-Cy5 nanoassemblies, enabling efficient FRET signal transduction. The platform further enables quantification of endogenous circRNA at the single-cell level and allows discrimination between breast cancer tissues and adjacent normal samples, highlighting its potential for high-resolution molecular diagnostics.

Building on these principles, He et al.^87^ developed the first tetrahedral DNA nanostructures (TDNs)-modified microfluidic chip for circRNA detection. In this integrated system, TDNs enable controlled spatial arrangement of BSJ-targeting probes along the polydimethylsiloxane (PDMS) microchannel surfaces, improving probe accessibility while reducing nonspecific adsorption and enhancing stability. Upon circRNA binding, a target-triggered isothermal HCR generates amplified fluorescence signals within the microfluidic channels. This platform achieves an ultrasensitive LOD of 19 a.m. under optimized conditions, while requiring only small sample volumes and maintaining stability over extended periods. Notably, it enables accurate quantification of circRNAs from cancer cell-derived RNA, with results closely matching RT-qPCR measurements (Figure 3C).

More recently, increasing efforts have been directed toward overcoming one of the major limitations of circRNA biosensing, namely the simultaneous detection of multiple clinically relevant targets. In this context, Zhang et al.^88^ developed a ligation-controlled single-molecule fluorescent biosensor for the multiplex measurement of the breast cancer-associated circRNAs: circFOXO3 and circMTO1. By integrating SplintR ligase-mediated BSJ recognition with EXPAR amplification and AuNP-based spherical nucleic acid nanoprobes, the platform achieved attomolar LODs of 8.34 a.m. and 9.84 a.m., respectively, while enabling simultaneous circRNA analysis in single cancer cells and successful discrimination between cancerous and healthy tissues (AUC = 1). Notably, the single-molecule imaging strategy combines high analytical sensitivity with specific BSJ recognition, demonstrating the potential of multiplex fluorescent biosensing for circRNA profiling in clinically relevant samples. Building on a different amplification strategy, Wang et al.^89^ demonstrated a synchronous 3D-DNA walking-driven nanodevice exploiting dual-color RNA aptamers activated by rolling circle transcription for label-free and simultaneous attomolar detection of circMTO1 and circCDYL. Importantly, the platform successfully discriminated breast cancer staging from early stage I–II (91.1%) to advanced stage III–IV (99.4%). Collectively, these platforms highlight that simultaneous multi-target circRNA detection in clinical tissue samples is achievable with single-molecule fluorescence approaches, though both currently rely on extracted RNA inputs rather than minimally processed biofluids.

In practical applications, achieving both high sensitivity and reproducibility remains challenging due to the stochastic nature of electromagnetic field distribution and the heterogeneous environment of real complex samples. To overcome these limitations, the formation of nanoscale junctions between metallic nanostructures plays a crucial role, as these regions enable strong electromagnetic coupling. However, rather than relying exclusively on pre-engineered substrates, emerging approaches exploit target-induced assembly processes to dynamically generate signal-active configurations. Among these strategies, core-satellite architectures enable the formation of multiple plasmonic junctions with controlled spatial arrangement. This approach was adopted by Xu et al.^90^ for the ultrasensitive detection of cancer-related circRNAs in serum samples, reliably distinguishing early-stage lung cancer (IA/IB) and its subtypes (IA1/IA3). A CHA-assisted magnetic SERS platform was developed based on the assembly of core-shell Au@MBA@Ag nanoprobes and Fe_3_O_4_@Ag magnetic nanoparticles. In this system, the formation of core-satellite structures generates multiple interparticle junctions, enabling strong electromagnetic coupling and the creation of highly active SERS hot spots. The analytical performance is further improved by magnetic enrichment, which facilitates target capture and concentration from complex matrices, and by using an internal standard for ratiometric calibration, reducing signal variability. As a result, the platform achieves ultrasensitive detection with a LOD as low as 5.5 a.m., significantly outperforming conventional tumor markers. This target-driven paradigm has also been extended by Xu et al.^91^ to intracellular SERS sensing (Figure 3D). In this approach, a dual-signal SERS nanoprobe based on a core-shell Au@4 MB N@Au structure was functionalized with a ROX-labeled DNA probe. The sensing mechanism relies on competitive hybridization between the target circRNA and the probe strands. Upon target binding, the reporter strand is displaced, increasing the distance with the nanoparticle surface and leading to a decrease in SERS intensity. Quantitative analysis is based on ratiometric measurement of the ROX signal relative to the internal standard 4 MB N, ensuring reliable signal normalization in the intracellular environment. The system exhibits high sensitivity, reaching a detection limit in the picomolar range, while maintaining good specificity toward the target. Application in living cells enabled quantitative monitoring of circRNA expression, with reduced SERS signals observed in lung cancer cells compared to normal cells, consistent with RT-PCR analysis.

As previously discussed, standard CRISPR possibilities enable precise genome editing through targeted DNA cleavage and repair pathway activation. Alternatively, these capabilities can be exploited to drive downstream signal transduction processes in novel configurations. This principle was applied by Liu et al.^92^ to achieve ultrasensitive circRNA detection at the attomolar level with a high signal-to-background response. In their platform, target recognition activates Cas13a-mediated cleavage of an rU-containing substrate probe, leading to the release of a trigger sequence that initiates PER cascades. These reactions generate numerous single-stranded G-quadruplex structures, which act as optically active signal units. The system achieves ultrasensitive circRNA detection down to 0.8 a.m. under controlled laboratory conditions, minimizes nonspecific background, and enables profiling in single breast cells and clinical tissue samples, demonstrating robustness in complex biological samples. Building on this strategy, the same group developed a more architecturally sophisticated mirror-synchronized asymmetric CRISPR-Cas12a nanoswitch for circRNA detection.^20^ The design introduces a dual-palindromic hairpin system (PHP1 and PHP2) that recognizes the BSJ and initiates an autonomous bidirectional polymerization-mediated amplification, generating abundant activator strands. These products subsequently trigger an asymmetric CRISPR-Cas12a cascade, where a full-sized crRNA induces primary *trans*-cleavage, followed by a split-crRNA-mediated secondary cleavage, enabling enhanced signal output. A Cy5/BHQ2-labeled probe converts Cas12a activity into a measurable optical signal. By decoupling target amplification from CRISPR activation and combining DNA nanotechnology with a multi-step CRISPR readout, this nanoswitch achieves ultrasensitive and rapid one-pot circRNA detection at the attomolar level (LOD: 1.07 a.m.), single-base BSJ discrimination, and single-molecule profiling in breast cancer cells and tissues.

Extending the circRNA sensing possibilities, a machine learning-assisted, polarity-switchable photoelectrochemical (PEC) biosensor has been developed to further improve signal reliability and support preliminary classification in circRNA-based diagnostic models.^93^ In this system, target-triggered amplification is coupled with Cu_2_O nanosphere-mediated photocurrent inversion, enabling dynamic switching between anodic and cathodic responses. This dual-mode signal output provides an internal validation mechanism that reduces susceptibility to background interference and enhances analytical robustness in complex matrices. In addition, the sensing interface is designed to be renewable, allowing repeated use of the electrode without compromising performance. This combined strategy enables ultrasensitive circRNA detection at the attomolar level in RNA extracted from whole blood samples, while achieving highly reliable discrimination between patients with cancer and healthy individuals in a limited proof-of-concept cohort.

Table 3 summarizes currently available circRNA biosensing strategies. The table highlights the diversity of detection platforms, including electrochemical, optical (fluorescent and SERS), colorimetric, FET, and PEC biosensors, along with their corresponding assay principles, target circRNAs, and biological matrices. A wide range of design strategies are employed to enhance sensitivity, from hybridization-based recognition to CRISPR-mediated amplification, including RCA, HCR, and enzyme-free cascades, with limits of detection often reaching the attomolar range. Notably, FET- and CRISPR-based electrochemical biosensors generally offer the fastest detection times, often operating within minutes and at room temperature, whereas amplification-heavy strategies such as RCA- or HCR-based fluorescence assays typically require longer assay times and elevated temperatures. In terms of sensitivity, CRISPR-integrated and SERS-based platforms achieve the lowest LODs, frequently in the attomolar range, outperforming conventional hybridization-only approaches. Additionally, the diversity of transducer surface modifications reflects efforts to optimize signal generation across different sensing modalities. Overall, variations in detection time, temperature requirements, and analytical performance underscore the trade-offs between rapid analysis and ultrasensitive detection, highlighting the ongoing evolution of circRNA biosensing technologies.

**Table 3:** Overview of circRNA biosensing strategies and analytical performance

Detection method	circRNA/disease	Amplification strategy	Surface functionalization	Assay time/temperature	LOD	Sample	Recovery	Reference
**Electrochemical biosensors**								

*Hybridization-based approaches*								
Signal-off PNA-based hybridization assay on an SPE/DPV readout	Circ-CDYL/HCC	AuNFs -enabled signal enhancement	CFME modified with AuNFs and thiolated PNA probes	7 h/RT	3.29 fM (Buffer)	human serum	N/R	Zhang et al.^76^
Signal-on DNA-based hybridization assay/ECL readout	hsa -circRNA-0137439/Bladder cancer	multiple signal amplification strategy, including RCA, quadruple amplification, and cyclic amplification	Fe-MOF@AuNPsmodified electrodes	5 h/up to 30°C	0.046 fM (0.1 M PBS pH 7.4 + 0.1 M TPrA)	human urine	93.3% - 118.81%	Zhang et al.^84^
*CRISPR-based approaches*								
Signal-off CRISPR/Cas13a-based assay using collateral cleavage of Td-MB complex on a gold electrode/DPV readout	bladder-cancer-associated circRNA/bladder cancer	CRISPR/Cas13a collateral cleavage-driven signal amplification	Td/MCH-modified gold electrode; MB redox intercalator	<10 min/RT	0.089 fM (Buffer)	human urine	N/R	Cheng et al.^85^
Signal-on CRISPR/Cas13a-based assay with MB-mediated signal transduction; SWV readout	cZNF292/AMI	PER-mediated cascade coupled with CRISPR/Cas12a collateral *trans*-cleavage; AuNPs-enabled signal enhancement	CB[7]@GE (PDDA/AuNPs/CB[7]) electrode; MB-DNA signal probe	1 h/RT	2.13 fM (Buffer)	whole blood	93.55% - 107.0%	Sha et al.^86^

**Optical biosensors**								

*Fluorescent biosensors*								
Signal on TDN- based hybridization on a microfluidic chip/Fluorescence intensity readout	circ_006127/Gastric cancer	hybridization chain reaction (HCR) signal amplification	tetrahedral DNA nanostructure (TDN)-modified PDMS microfluidic chip	1 h/RT	19 a.m. (Buffer)	total RNA extracted from cancer cells (AGS cells)	N/R	Nielsen et al.^36^
QD-Cy5 FRET modulation/Fluorescence intensity readout	circMTO1/BC	TWJ-mediated cascade amplification (SDA + PG-RCA)	DNA three-way junction-guided functionalization of quantum dots	3 h/up to 95°C	7.12 a.m. (Buffer)	spiked human serum (1% and 5%)	98.1% - 104.3%	Liu et al.^79^
Signal-on detection via FRET hairpin probe activation/Fluorescence intensity readout	circTEX2/HCC	dual nucleic-acid amplification via RT-RCA coupled with netlike HCR	N/A	5 h/up to 85°C	0.1 pM (Buffer)	extracted cellular RNA from cell lysates	N/R	Dong et al.^94^
Ligation-controlled single-molecule biosensor/TIRF single-molecule imaging readout	circFOX3, circMTO1/Breast cancer	SplintR ligase-mediated ligation coupled with EXPAR-induced DNAzyme generation	AuNP-based spherical nucleic acid nanoprobe	2 h/up to 55°C	8.34 a.m. circFOX3; 9.84 a.m. circMTO1 (Buffer)	extracted RNA from cancer cells and clinical breast tissue samples	N/R	Zhang et al.^88^
Dual-color RNA aptamer lighting-up biosensor/Fluorescence intensity readout	circMTO1, circCDYL/Breast cancer	DSN-powered 3D-DNA walking coupled with T7/T3 RNA polymerase-catalyzed RCT	streptavidin-coated magnetic bead-capture probe conjugates	3 h/up to 60°C	10.96 a.m. circMTO1; 17.78 a.m. circCDYL (Buffer)	extracted RNA from breast cancer cells; spiked human serum (10%)	97.90–99.17% circMTO1;97.60–98.67% circCDYL (Serum)	Wang et al.^89^
*SERS biosensors*								
Signal-on DNA-based hybridization/Ratiometric Raman readout	circVAPA/Lung cancer	CHA-mediated enzyme-free cyclic amplification	magnetic core-satellite SERS platform based on Fe_3_O_4_@Ag and Au@Ag nanostructure	2 h/37°C	5.5 a.m. (Buffer)	human serum	92.1% – 101.3%	Xu et al.^90^
3Signal-off DNA-based competitive hybridization/Ratiometric Raman readout	circSATB2 (BSJ)/Lung cancer	N/A	dual-signal Au@4 MB N@Au SERS nanoprobes with embedded internal Raman standard	24 h/37°C	0.043 pM (Buffer)	extracted cellular RNA from cell lysates	95.6% – 101.6%	Xu et al.^91^
*CRISPR-based biosensors*								
Mirror-synchronized asymmetric CRISPR nanoswitch realizing Cy5 photon/Camera sensor detecting and quantifying single photon events	circMTO1/breast cancer	dual-palindromic hairpin-mediated exponential enrichment coupled with asymmetric CRISPR/Cas12a *trans*-cleavage cycling	N/A	25 min/37°C	1.07 a.m. (Buffer)	extracted RNA from human breast cancer cells	95.9% - 103.0%	Liu et al.^20^
Thioflavin T light-up fluorescence via G-quadruplex formation/Fluorescence intensity readout	circHIPK3/breast cancer	CRISPR/Cas13a-mediated *trans*-cleavage triggering PER cascade amplification	N/A	40 min/37°C	0.83 a.m. (Buffer)	total RNA extracted from cancer cell lines and clinical breast tissue samples	N/R	Liu et al.^92^
*Colorimetric biosensors*								
Formation of G-Quadruplex-Hemin DNAzymeDNAzyme-catalyzing TMB oxidation/Absorbance readout	cSMARCA5/HCC	CHA cascade coupled with G-quadruplex-hemin DNAzyme amplification	MNPs functionalized with BSJ-specific hairpin probes	3.5 h/up to 80°C	1.93 fM (Buffer)	whole blood	N/R	Li et al.^95^

**FET biosensors**								

Label-free DNA probe-modified nanoribbon chip/electronic readout	circNFIX/glioma	N/A	SOI nanoribbon FET chip functionalized with complementary DNA probes	<10 min/RT	0.011 fM (Buffer)	extracted plasma RNA	N/R	Ivanov et al.^96^

**PEC biosensors**								

Polarity-switchable/PEC readout	circSATB2/lung cancer	TWJ–Exonuclease III–assisted cyclic amplification	CdS/T-COF photoactive layer, DNA probes and Cu_2_O nanospheres	2 h/37°C	7.6 a.m. (Buffer)	extracted whole-blood RNA	96.2% - 101.8%	Geng et al.^93^
Photocurrent quenching/PEC readout	circSATB2/lung cancer	CRISPR/Cas13a *trans*-cleavage-triggered HCR and dsDNA-templated *in situ* Cu nanocluster generation	Z-scheme T-COF/Ag_2_S photoactive composite on ITO electrode/Cu nanoclusters as photocurrent quencher	4 h/37°C	0.5 fM (Buffer)	extracted whole-blood RNA	98.2% - 103.1%	Yuan et al.^97^

**AMI:** acute myocardial infarction; **AuNPs:** gold nanoparticles; **AuNFs:** gold nanoflowers; **BC:** breast cancer; **BSJ:** back-splice junction; **CB[7]:** cucurbit[7]uril; **CdS:** cadmium sulfide; **CFME:** carbon fiber microelectrode; **CHA:** catalytic hairpin assembly; **DPV:** differential pulse voltammetry; **DSN:** duplex-specific nuclease; **ECL:** electrochemiluminescence; **EXPAR:** exponential amplification reaction; **Fe-MOF:** iron-based metal-organic framework; **FET:** field-effect transistor; **FRET**: Förster resonance energy transfer; **HCC:** hepatocellular carcinoma; **HCR:** hybridization chain reaction; **ITO:** indium tin oxide; **MB:** methylene blue; **MCH:** 6-mercapto-1-hexanol; **MNPs:** magnetic nanoparticles; **N/A:** not applicable; **N/R:** not reported; **PEC:** photoelectrochemical; **PDDA**: poly(diallyldimethylammonium chloride); **PDMS:** polydimethylsiloxane; **PER:** primer exchange reaction; **PG-RCA**: primer generation–rolling circle amplification; **PNA**: peptide nucleic acid; **QD:** quantum dot; RCA: rolling circle amplification; **RCT:** rolling circle transcription; **RT-RCA:** reverse transcription–rolling circle amplification; **SDA:** strand displacement amplification; **SERS:** surface-enhanced Raman scattering; **SOI:** silicon-on-insulator; **SPE:** screen-printed electrode; **SplintR ligase:** chlorella virus DNA ligase; **SWV:** square-wave voltammetry; **T-COF:** two-dimensional covalent organic framework; **Td:** tetrahedral DNA; **TDN:** tetrahedral DNA nanostructure; **TIRF:** total internal reflection fluorescence; **TMB:** 3,3′,5,5′-tetramethylbenzidine; **TPrA:** tripropylamine; **TWJ:** three-way junction.

Indicative examples of the circRNA biosensing platforms reported to date are being summarized (electrochemical, optical (fluorescent and SERS), colorimetric, field-effect transistor (FET), and photoelectrochemical (PEC). For each system, the detection method, targeted circRNA and associated disease, biological sample, amplification strategy, and surface functionalization are reported alongside key analytical parameters, including assay time, operating temperature, LOD, and recovery values were applied.

## Challenges and clinical translation

Despite substantial progress in circRNA biosensing, it is important to recognize that the majority of currently reported platforms remain at the proof-of-concept stage. Most systems have been evaluated under highly controlled experimental conditions using synthetic targets, spiked samples, or extracted RNA, whereas validation in minimally processed clinical specimens has been reported only in a limited number of studies, often involving relatively small and highly selected patient cohorts. Consequently, the remarkable analytical performances frequently reported, including femtomolar and attomolar LOD, should be interpreted within the context of the experimental conditions under which these platforms were developed. Beyond analytical sensitivity, several challenges continue to limit routine clinical translation. A major bottleneck is achieving high analytical specificity in complex biological matrices, where distinguishing circular from linear RNA isoforms remains technically demanding, especially in low-abundance clinical samples. Matrix effects, nonspecific adsorption, and sequence similarity can further compromise reliable detection and quantification.^98^

Proteins and lipids present in biofluids can adsorb non-specifically onto biosensor surfaces, altering probe accessibility, blocking active sites, and increasing background signal, a phenomenon commonly referred to as biofouling. Moreover, although circRNAs themselves are intrinsically resistant to degradation, endogenous nucleases may compromise probe components or amplification intermediates, thereby reducing assay sensitivity and reproducibility. Protein-rich environments, high ionic strength, and pH variability in clinical samples can further perturb hybridization thermodynamics, affect CRISPR effector activity, and alter electrochemical or optical signal baselines. Additionally, the co-presence of homologous linear RNA isoforms sharing identical sequences except at the BSJ represents a persistent specificity challenge, particularly in matrices where these transcripts are abundant relative to the circular form. To mitigate these effects, several strategies have been explored.^99^^,^^100^ Surface passivation using blocking agents such as mercaptohexanol, bovine serum albumin, or polyethylene glycol can reduce nonspecific adsorption and improve signal-to-noise ratios on electrochemical and SERS platforms. Magnetic enrichment approaches, as employed in core-satellite SERS systems, enable target concentration and partial matrix simplification prior to detection.^101^^,^^102^ Likewise, ratiometric signal readout strategies provide built-in normalization against matrix-induced signal fluctuations. Nevertheless, RNA extraction or enrichment prior to sensing remains the most widely adopted approach to reduce matrix interference, as reflected by the high proportion of platforms summarized in Table 3 that rely on purified RNA inputs. Looking forward, the development of biosensors capable of operating directly in minimally processed or unprocessed clinical samples, without prior RNA extraction, will be essential to fully exploit the POC potential of circRNA biosensing technologies.

Standardization and reproducibility are also major concerns. Differences in probe design, surface functionalization, amplification chemistry, and signal transduction strategies often lead to inconsistent analytical performance across platforms. In addition, validation across independent laboratories and fabrication batches remains largely unexplored, while the field still lacks universally accepted calibration standards and validation workflows.^103^ Another important challenge is multiplexed detection. For cancer and other heterogeneous diseases, clinically useful assays will likely need to quantify panels of circRNAs rather than single biomarkers. Although multiplexing is increasingly being explored, most current biosensor systems remain focused on one target at a time, which limits their diagnostic and prognostic utility.^104^

**Figure 4:**
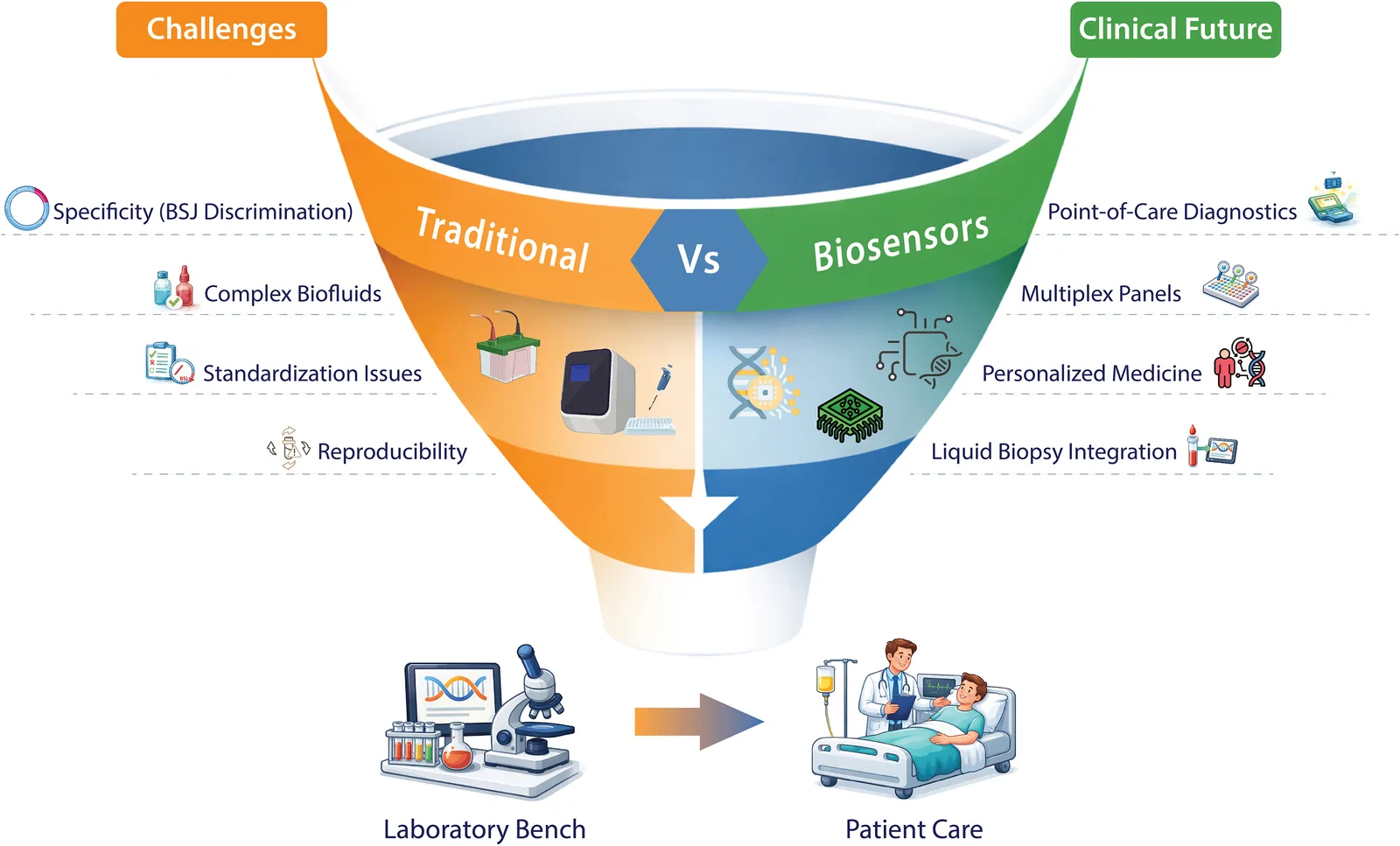
Challenges and clinical translation pathways for circRNA detection technologies Schematic overview of the main challenges and future directions in the clinical implementation of circRNA detection. Traditional approaches face limitations related to specificity in complex biological matrices, standardization, reproducibility, and regulatory barriers. In contrast, emerging biosensor-based strategies offer opportunities for point-of-care diagnostics, multiplexed analysis, personalized medicine, and integration with liquid biopsy. The transition from laboratory research to clinical application highlights the potential of biosensors to overcome current limitations and enable clinically relevant circRNA-based diagnostics

From a translational perspective, biosensor-based methods must also be considered alongside established techniques such as RT-qPCR and sequencing (Figure 4). Biosensors offer clear advantages in speed, miniaturization, and point-of-care potential, but conventional approaches still provide stronger standardization, broader clinical validation, and deeper familiarity within diagnostic practice. Furthermore, the clinical relevance of many proposed circRNA targets remains to be confirmed in appropriately powered patient cohorts, and the ability of biosensor-derived measurements to provide actionable information within existing diagnostic and monitoring workflows has yet to be fully established. As a result, the current gap between promising research prototypes and clinically deployable diagnostics remains substantial. Nevertheless, the growing body of proof-of-concept studies highlights the considerable potential of biosensor technologies and provides a strong foundation for future clinical translation.

## Conclusions and future perspectives

circRNAs represent a compelling class of biomolecules whose intrinsic stability and disease-specific expression profiles position them as promising candidates for next-generation diagnostics and prognostics. As highlighted throughout this review, circRNAs are involved in diverse pathological processes, including cancer progression, immune modulation, and infectious diseases, and can be detected in clinically relevant biofluids, supporting their application in minimally invasive diagnostic strategies. Furthermore, the field is increasingly transitioning from single-marker evaluations to multivariate circRNA signatures. This shift is exemplified by specialized panels capable of predicting immunotherapy efficacy^105^ via immune infiltration profiling, differentiating specific viral etiologies from closely related infectious agents,^106^ and stratifying early-stage malignancies.^107^ Collectively, these developments demonstrate that multi-target panels, rather than isolated biomarkers, are essential to achieve the statistical power required for robust clinical classification.

At the same time, advances in analytical methods, including the development of biosensor-based platforms, have expanded the available tools for their detection and quantification. Despite these conceptual and analytical advances, the clinical translation of circRNAs remains severely bottlenecked by fundamental technical and methodological hurdles. Notably, accurately discriminating circular transcripts from their linear counterparts sharing identical sequence exons remains technically fraught,^108^ particularly when profiling low-abundance targets in complex biological matrices. In addition, variability across analytical platforms, lack of standardized workflows spanning sample collection, RNA extraction, and data normalization, and difficulties in reliable quantification hinder reproducibility and large-scale validation. Consequently, the literature is saturated with fragmented, low-powered discoveries that fail validation in large, independent cohorts, a deficit compounded by a lack of rigorous digital quantification standards. These analytical inconsistencies, paired with a lack of clear regulatory frameworks for non-coding RNA diagnostics, “currently preclude the integration of circRNA assays into routine clinical workflows.

To bridge the gap between benchtop discovery and clinical implementation, the field must move past redundant biomarker identification and prioritize architectural and analytical integration. Looking forward, the integration of highly sensitive detection systems with multiplexed and data-driven approaches is expected to accelerate the clinical adoption of circRNAs. In this context, future efforts should focus on improving detection specificity in complex biological matrices, establishing standardized and reproducible workflows, and addressing regulatory requirements for clinical translation. Importantly, resolving the heterogeneity of complex diseases like cancer will require a shift from qualitative marker discovery to quantitative, multiplexed systems biology capable of simultaneously profiling heterogeneous molecular patterns. The development of multiplexed circRNA panels, combined with advances in biosensing technologies, microfluidic integration, and computational analysis, is expected to enhance diagnostic accuracy and enable more comprehensive disease profiling.^104^^,^^109^^,^^110^

Ultimately, bridging the gap between analytical development and clinical application will require coordinated interdisciplinary efforts and rigorous validation in large patient cohorts. In this context, the rational selection of circRNA targets and analytical strategies will be critical to maximize clinical impact. With continued technological and clinical advancements, circRNAs are poised to become key components of next-generation precision diagnostics and prognostic tools.

## Acknowledgments

S.C. acknowledges AIRC under MFAG 2022−ID. 27586 project. A.G. acknowledges the PhD program in Clinical and Translational Oncology (CTO) at Scuola Superiore Meridionale (SSM) funded under Investment I.3.3, PNRR (Piano Nazionale di Ripresa e Resilienza) Scholarships for innovative PhD programs addressing the innovation needs of industry. P.M.K. and A.M. acknowledge 10.13039/501100004710Fondazione Umberto Veronesi Postdoctoral Fellowship 2026. G.I. acknowledges the PhD program in Genomic and Experimental Medicine (10.13039/100017673GEM) at Scuola Superiore Meridionale (SSM). This project has received funding from the European Union’s 10.13039/501100007601Horizon 2020 research and innovation program under the Marie Skłodowska-Curie grant no. 101110684 SMART - Sima Singh.

## Author contributions

A.G.: writing – original draft. P.M.K.: writing – original draft, supervision, and conceptualization. A.M.: writing – original draft. G.I.: writing – original draft. S.S.: writing – original draft. M.D.L.: editing and supervising. S.C.: writing – original draft, supervision, project administration, funding acquisition, and conceptualization.

## Declaration of interests

The authors declare no competing interests.
